# Cerebellar transcranial direct current stimulation for learning a novel split-belt treadmill task: a randomised controlled trial

**DOI:** 10.1038/s41598-020-68825-2

**Published:** 2020-07-16

**Authors:** Nitika Kumari, Denise Taylor, Usman Rashid, Alain C. Vandal, Paul F. Smith, Nada Signal

**Affiliations:** 1 Health and Rehabilitation Research Institute, Auckland University of Technology, Auckland, New Zealand; 2 Department of Statistics, University of Auckland, Auckland, New Zealand; 3 Department of Pharmacology and Toxicology, School of Biomedical Sciences, Brain Health Research Centre, University of Otago, Dunedin, New Zealand; 4 Brain Research New Zealand, Auckland, New Zealand

**Keywords:** Therapeutics, Randomized controlled trials

## Abstract

This study aimed to examine the effect of repeated anodal cerebellar transcranial direct current stimulation (ctDCS) on learning a split-belt treadmill task. Thirty healthy individuals randomly received three consecutive sessions of active or sham anodal ctDCS during split-belt treadmill training. Motor performance and strides to steady-state performance were evaluated before (baseline), during (adaptation), and after (de-adaptation) the intervention. The outcomes were measured one week later to assess absolute learning and during the intervention to evaluate cumulative, consecutive, and session-specific effects. Data were analysed using linear mixed-effects regression models. During adaptation, there was no significant difference in absolute learning between the groups (*p* > 0.05). During de-adaptation, a significant difference in absolute learning between the groups (*p* = 0.03) indicated slower de-adaptation with anodal ctDCS. Pre-planned secondary analysis revealed that anodal ctDCS significantly reduced the cumulative (*p* = 0.01) and consecutive-session effect (*p* = 0.01) on immediate adaptation. There were significant cumulative (*p* = 0.02) and session-specific effects (*p* = 0.003) on immediate de-adaptation. Repeated anodal ctDCS does not enhance motor learning measured during adaptation to a split-belt treadmill task. However, it influences the maintenance of learnt walking patterns, suggesting that it may be beneficial in maintaining therapeutic effects.

## Introduction

Cerebellar transcranial direct current stimulation (ctDCS), a non-invasive brain stimulation technique, has the potential to become a neuro-rehabilitation tool to facilitate therapy-induced recovery in people with brain lesions^1,2^. Various neuro-physiological studies^3–5^ have demonstrated that ctDCS is capable of altering the excitability of the cerebellum, a critical structure in error-driven motor learning^6–8^. There is evidence of improved gains in motor performance up to 48 h after a single application of anodal ctDCS^9,10^. However, evidence of its ability to induce long-lasting changes in motor performance with multiple sessions of stimulation is limited^11,12^. This study aimed to elucidate the effect of three consecutive sessions of anodal ctDCS on motor learning of a novel treadmill walking task in healthy individuals.

Motor learning is an internal process associated with practice or experience, which results in the long-lasting acquisition of skilled motor performance^13^. To examine the effect of an intervention on motor learning, evaluation of motor performance more than 24 h after the intervention is required^13^. This is because the transient effects of the intervention dissipate, but the relatively permanent effects remain at the follow-up evaluation reflecting learning. Such learning is fundamental for acquiring new motor skills and adapting to changing environments in our daily lives. Motor learning is commonly investigated in the laboratory through motor skill and motor adaptation paradigms. Motor skill training paradigms often use novel or complex motor skills, which may take weeks or months to master. In contrast, motor adaptation paradigms involve modifying a well-learnt movement in response to error signals (adaptation) and are characterised by the persistence of adapted patterns upon removal of the error (after-effects) that gradually returns to its baseline pattern over time (de-adaptation)^14^. Often a person adapts to the induced error within minutes to hours^15^. However, with repeated exposure to errors, immediate reductions in errors^16^, faster adaptation on subsequent exposures^17^, and a decrease in after-effects are observed^16^.

There is a growing body of evidence investigating the effect of ctDCS delivered via a positively charged electrode (anode) or a negatively charged electrode (cathode) on modulating motor performance in healthy individuals. While the majority of the studies have investigated motor skill paradigms, there are limited studies that have evaluated the effect of anodal ctDCS on learning of motor adaptation tasks, particularly functional tasks such as walking (locomotor adaptation)^18^. In addition, research investigating the effect of ctDCS on learning motor adaptation tasks has failed to evaluate its effect on motor learning measured for more than 24 h after the intervention^19–22^. Only one study has evaluated the effect of anodal ctDCS on locomotor adaptation tasks involving walking on a split-belt treadmill in healthy individuals^20^. The authors demonstrated that a single session of anodal ctDCS enhanced the rate of adaptation during a split-belt treadmill task compared to cathodal and sham stimulation. As this was investigated within a single session, it is still unknown whether the effects of ctDCS accumulate over multiple sessions, or whether ctDCS can modulate long-lasting acquisition of a locomotor adaptation task measured after a delay of days or weeks. The primary goal of this study was to investigate the effects of three consecutive sessions of anodal ctDCS on learning a split-belt treadmill walking task in healthy individuals after a delay of one week. Additionally, we investigated if the effects were cumulative over the three sessions of the intervention, including the consecutive and session-specific effects.

## Methods

### Design and setting

This study was a single-centre, double-blinded, parallel, randomised, sham-controlled design. Data were collected in a movement analysis laboratory based at Auckland University of Technology’s Millennium Institute in Auckland, New Zealand. Participants were randomised with a 1:1 ratio to either the active or the sham ctDCS group using a pseudo-random number generator in MATLAB 2015a (MathWorks Inc.). All participants and the principal investigator, who administered the ctDCS application and measured the outcomes, were blinded to group allocation. To ensure blinding, two separate battery-operated stimulators were pre-programmed as either active or sham and labelled with separate codes by another researcher. This researcher was also involved with the generation of the random allocation sequence, enrolment, and allocation of participants to interventions. Blinding was maintained until the completion of the data analysis. The study was registered retrospectively on 5th August 2019 with the Australian New Zealand Clinical Trials Registry (Registration Number ACTRN12619001074189).

### Study participants

The sample size for this study was estimated based on a previous research^23^. This was due to a lack of data describing the effects of ctDCS on motor learning after a delay of one week in motor adaptation tasks, and insufficient reporting in studies investigating the effects of ctDCS on motor learning in motor skill tasks to calculate the sample size. The sample size was elevated to 30 participants to allow for sampling error and dropouts. This sample size is larger than all the previous studies that have reported enhanced gains in motor adaptation tasks with a single session of ctDCS^20,24,25^. Participants were recruited through posters on university notice boards and word-of-mouth. Participants were included if they were healthy individuals aged 18 years or above. Exclusion criteria consisted of a history of orthopaedic, cardiac, or neurological conditions that could interfere with walking, and any contraindications to the application of ctDCS^26^. Eligible participants volunteered to participate in the study by giving informed written consent, which conformed to the Declaration of Helsinki. The study was approved by the Ethics Committee of the Auckland University of Technology (16/338).

### Study procedure

**Figure 1:**
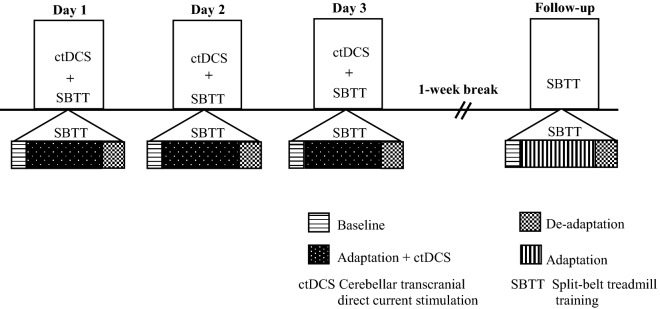
An illustration of the study protocol.

Each participant attended four sessions; three intervention sessions held on consecutive days and a follow-up session one week later (see Fig. 1 for an illustration of the study protocol). During the intervention sessions, participants received either active ctDCS or sham ctDCS during split-belt treadmill walking according to the randomisation schedule. At follow-up, split-belt treadmill walking was undertaken without ctDCS.

### Intervention

**Figure 2:**
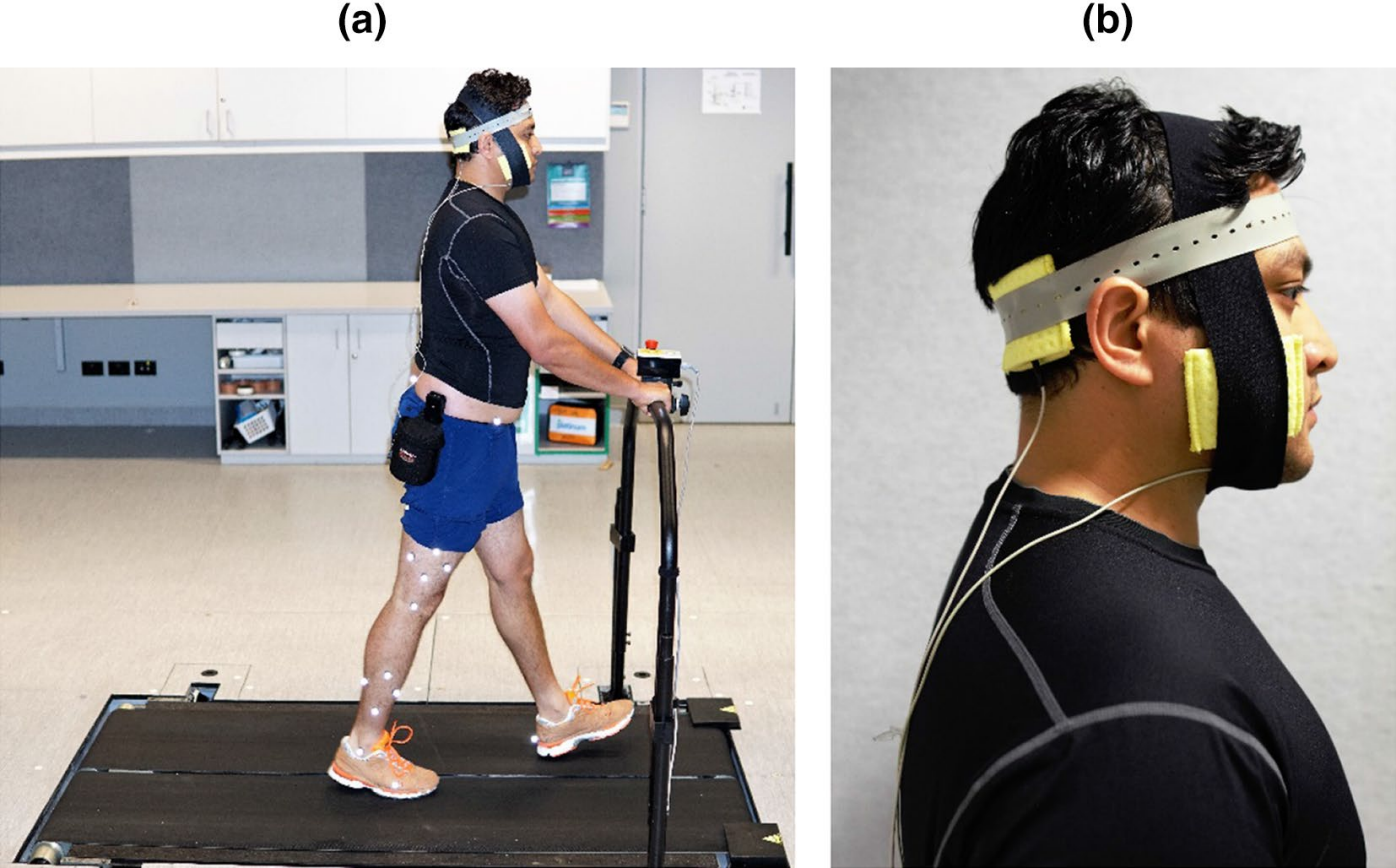
Overview of the experimental setup. (**a**) Participant performing the split-belt treadmill walking task, (**b**) ctDCS electrode positioning.

Cerebellar tDCS (HDCstim part of HDC kit, Magstim) was delivered via two electrodes (25 cm^2^) embedded in 0.9% saline-soaked sponges. The anodal electrode was placed 3 cm lateral to the inion to position it over the cerebellar hemisphere ipsilateral to the dominant leg, which was placed on the fast belt of the split-belt treadmill^20^. The cathode was placed over the ipsilateral buccinator muscle (see Fig. 2 for an overview of the experimental setup on one of the participants who gave informed consent to publish his photograph in an online open-access publication). The active ctDCS stimulator delivered 2 mA of current for 15 min with a 30-s ramp-up and ramp-down duration to slowly attenuate skin sensation^27,28^. The sham ctDCS stimulator ramped up the current to 2 mA over 30 s and then immediately ramped down to 0 mA over 30 s to ensure effective blinding. Participants in both the groups were familiarised with the ctDCS sensation by turning on the stimulator for a few seconds before the start of the treadmill walking task. To monitor any adverse event after ctDCS application, each participant was asked to give their feedback regarding the sensation of the ctDCS.

### Split-belt treadmill walking task

The split-belt treadmill task involved walking on a split-belt treadmill (Bertec Corporation, USA) in three phases: baseline, adaptation, and de-adaptation. Each phase was defined by belt speed (slower, faster) and belt symmetry (belts moving together) or asymmetry (belts moving at different speeds). Participants were instructed to stand in the middle of the treadmill with one foot on each belt holding onto a front rail, looking straight ahead whilst walking. The participant’s fastest comfortable walking speed was assessed by slowly increasing treadmill speed until the participant reported an inability to comfortably tolerate any further increase. This was undertaken three times and averaged to determine the speed of the faster treadmill belt. The speed of the slower belt was set to half that of the fast belt^29^. During *baseline,* participants walked with symmetrical belts at the slow gait speed. After 2 min of walking, the treadmill was stopped, and the ctDCS unit was turned on. During the *adaptation* phase, the treadmill was restarted at the slow gait speed, and then the belt speed of the fast belt was increased until the fast gait speed was attained. This asymmetrical speed ratio of 2:1 was then maintained for 15 min. Finally, in the *de-adaptation* phase, the ctDCS was turned off, and the participant walked for 10 min with both belts symmetrical at the slow speed (see Fig. 1 for an illustration of the study protocol showing split-belt treadmill walking task).

### Data collection

A Vicon motion capture system (Vicon Nexus 2.4, Vicon Motion System Inc.) was used to record force and position data from treadmill force plates and reflective markers, respectively. The position of 33 reflective markers, placed according to the Cleveland clinic model^30^, was captured via nine-cameras at a frame rate of 200 Hz. The data were recorded during the last minute of the baseline phase and throughout the adaptation and the de-adaptation phase.

### Outcome measures

Motor performance was measured based on step length symmetry. Step length symmetry is a kinematic variable under predictive control, which demonstrates robust adaptation during split-belt treadmill walking^15^. It is calculated as:

1 $$Step\,\, length \,\,symmetry = \frac{fast \,\,leg \,\,step \,\,length - slow \,\,leg\,\, step\,\, length}{{fast \,\,leg \,\,step\,\, length + slow \,\,leg\,\, step\,\, length}}$$

For Eq. (1), a symmetry value of zero represents perfect symmetry, whereas a positive value indicates a longer fast leg step length and a negative value indicates a longer slow leg step length^20^. Each step length was calculated as the antero-posterior distance between the ankle marker of each leg at the heel strike of the leading leg^20^.

**Figure 3:**
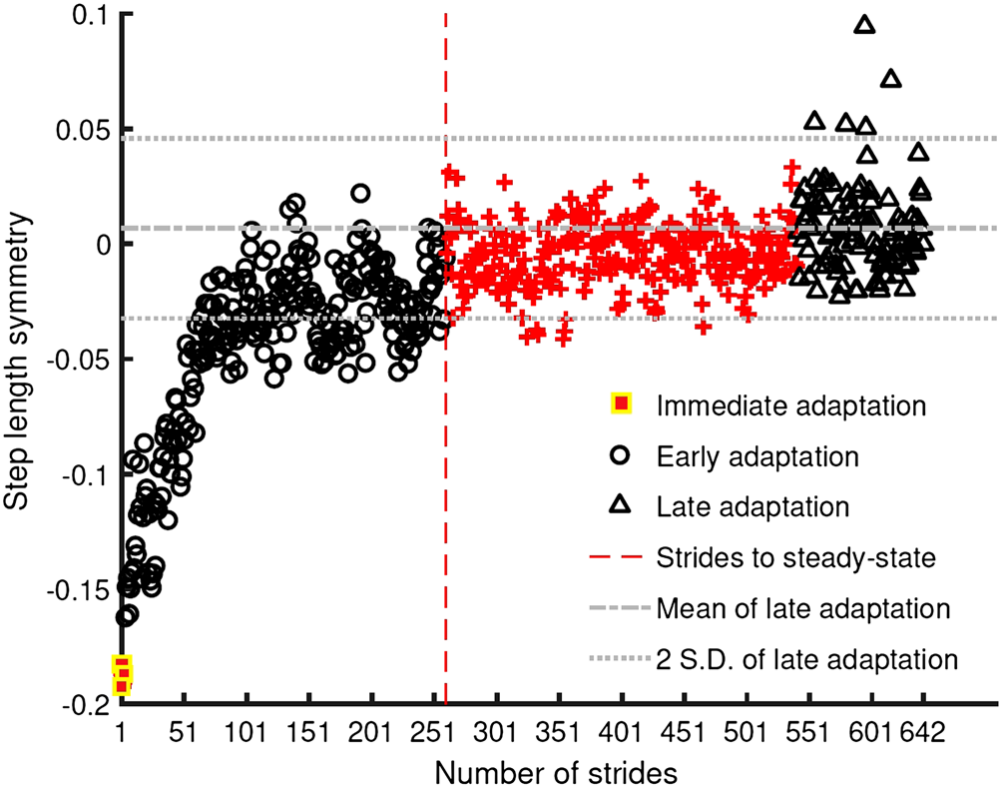
Symmetry series from a single participant during the adaptation phase illustrating how motor performance (immediate, early and late) and strides to steady-state were calculated for each participant.

Motor performance was determined by measuring mean step length symmetry during specified time periods during the adaptation and de-adaptation phases:

Immediate Adaptation and Immediate De-adaptation (represented as yellow squares in Fig. 3) were determined from the mean step length symmetry of the first three strides of the adaptation and de-adaptation phases, respectively.

Early Adaptation and Early De-adaptation (represented as black circles in Fig. 3) were determined from the mean step length symmetry of the fourth stride to the point where steady-state performance was achieved for individual participants during the adaptation and de-adaptation phases.

Late Adaptation and Late De-adaptation (represented as black triangles in Fig. 3) were determined from the mean step length symmetry of the last 100 strides of the adaptation and de-adaptation phase, respectively. The last 100 strides were used to represent late adaptation as it was utilised in the calculation of strides to steady-state motor performance explained below.

The strides to steady-state motor performance was calculated using an existing method from the split-belt treadmill literature^31^. This method marks the strides to steady-state of a participant using their symmetry series. The first stride (represented as a red vertical dashed line in Fig. 3) from a 30-stride bin was considered as strides to steady-state if the mean of the bin was within two standard deviations of the mean of the last 100 strides. Thus, the performance of an individual is tied to the variability of their response in addition to the rate of decay of their symmetry series. Refer to Fig. 3 for an illustration of all the above outcome measures in the symmetry series of a single participant.

### Data processing

A MATLAB-based implementation of the Detection and Correction Algorithm (DACA)^32^ was utilised for determining the gait events: heel-strikes and toe-offs for each foot. Once the gait events were detected, fast step length and slow step length were calculated^20^.

### Statistical analysis

**Figure 4:**
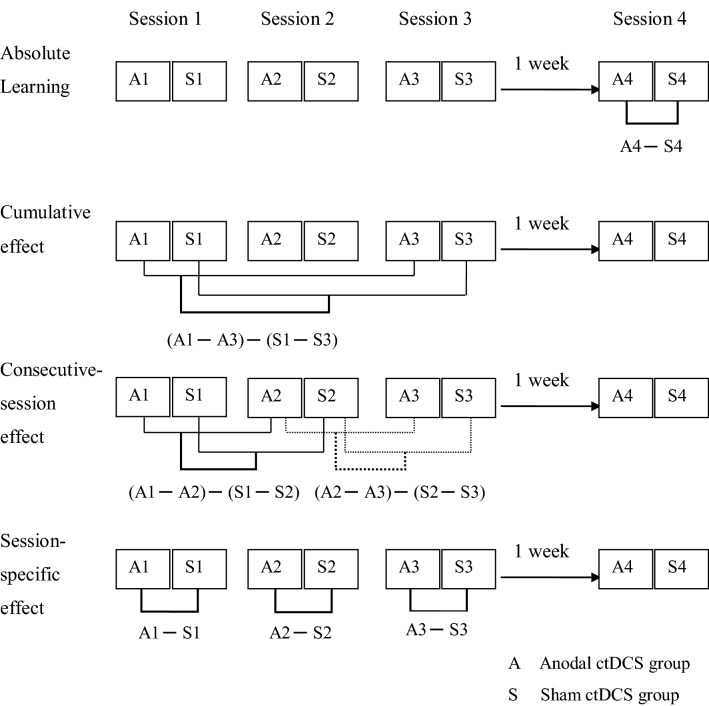
Statistical analysis for absolute learning, cumulative effect, consecutive-session effects and session-specific effect measurement time points.

The statistical analysis aimed at answering the following questions: (1) what are the absolute learning, cumulative, consecutive-session, and session-specific effects of multi-session anodal ctDCS on the adaptation phase of split-belt treadmill walking? (2) What are the absolute learning, cumulative, consecutive-session, and session-specific effects of multi-session anodal ctDCS on the de-adaptation phase of split-belt treadmill walking? These questions were addressed with respect to *motor performance* and *strides to steady-state motor performance*. Motor performance was analysed with respect to (a) immediate; (b) early; and (c) late adaptation and de-adaptation, respectively (see Fig. 4 for an illustration of statistical analysis performed).

Linear mixed regression analyses in R (R Foundation for Statistical Computing version 3.5.0) were used to answer these questions. The lme4 package version 1.1–17 was used for fitting all models^33^. The linear mixed regression method was chosen in preference to repeated measures ANOVAs because of the problems caused by extensive correlation in repeated measures data, where the ANOVA assumption of sphericity is often violated^34–37^. Instead, the linear mixed regression method models the covariance matrix structure of the repeated measures data, using information criteria such as the Akaike’s Information Criterion (AIC) as an indicator of the best model (the smaller, the better)^34–37^. The AIC and Bayesian Criterion (BIC) are the most commonly used information criteria used to assess model fit; however, the AICc (a corrected version of Akaike’s Information Criterion) is thought to be superior for small sample sizes^37^. In addition, linear mixed regression analysis yields higher statistical power compared to repeated measures ANOVAs and reduces the risk of type-I error^38^. One of the many advantages of linear mixed model analysis within the generalized linear mixed model (GLMM) framework is that ‘link’ functions can be used for non-normal distributions^34–37^. We were aware that non-parametric, rank-based, statistical analysis (e.g., Brunner et al.^39^) was an option, but this was not considered because the distributional assumptions (e.g., normality, homogeneity of variance) of the linear mixed regression method were either fulfilled (see below) or a different distribution (i.e. gamma distribution) was used. A blinded a priori co-variate selection was undertaken to identify co-variates explaining 5% or more of the variance in the data. These covariates were added to the models to control for their effect. As testing group differences at baseline is considered invalid^40^, we entered mean baseline symmetry from session 1 as a covariate to the models where it explained more than 5% or more of the variance in the data. This also catered to any difference in the mean baseline symmetry of individual groups from perfect symmetry. All the models included a random intercept effect for participants to account for the between-participant variance in the data. Model selection was based on least AICc values^41^, which indicates how well the covariance model fits the repeated measures^34,36,42^. Hypothesis testing was undertaken by entering participant and group as categorical variables and session, mean step length symmetry, and strides to steady-state as continuous variables. All the data were tested to fit the linear mixed model, which assumes that its residuals are normally distributed, and the variance of the residuals is homogeneous across the fitted values. In the case of data variables for which this model failed, models with alternative distributions and link functions were evaluated with AICc^34–37^. Therefore, a Gaussian (i.e., normal) distribution was used to model the data pertaining to motor performance and a Gamma distribution with a log link was used to model the data pertaining to strides to steady-state motor performance^43^.

## Results

Thirty participants were recruited to the study between November 2016 and January 2017 (See study flow and baseline demographic characteristics in supplementary file). All participants completed the research protocol and intervention without any adverse events or protocol deviations.

**Figure 5:**
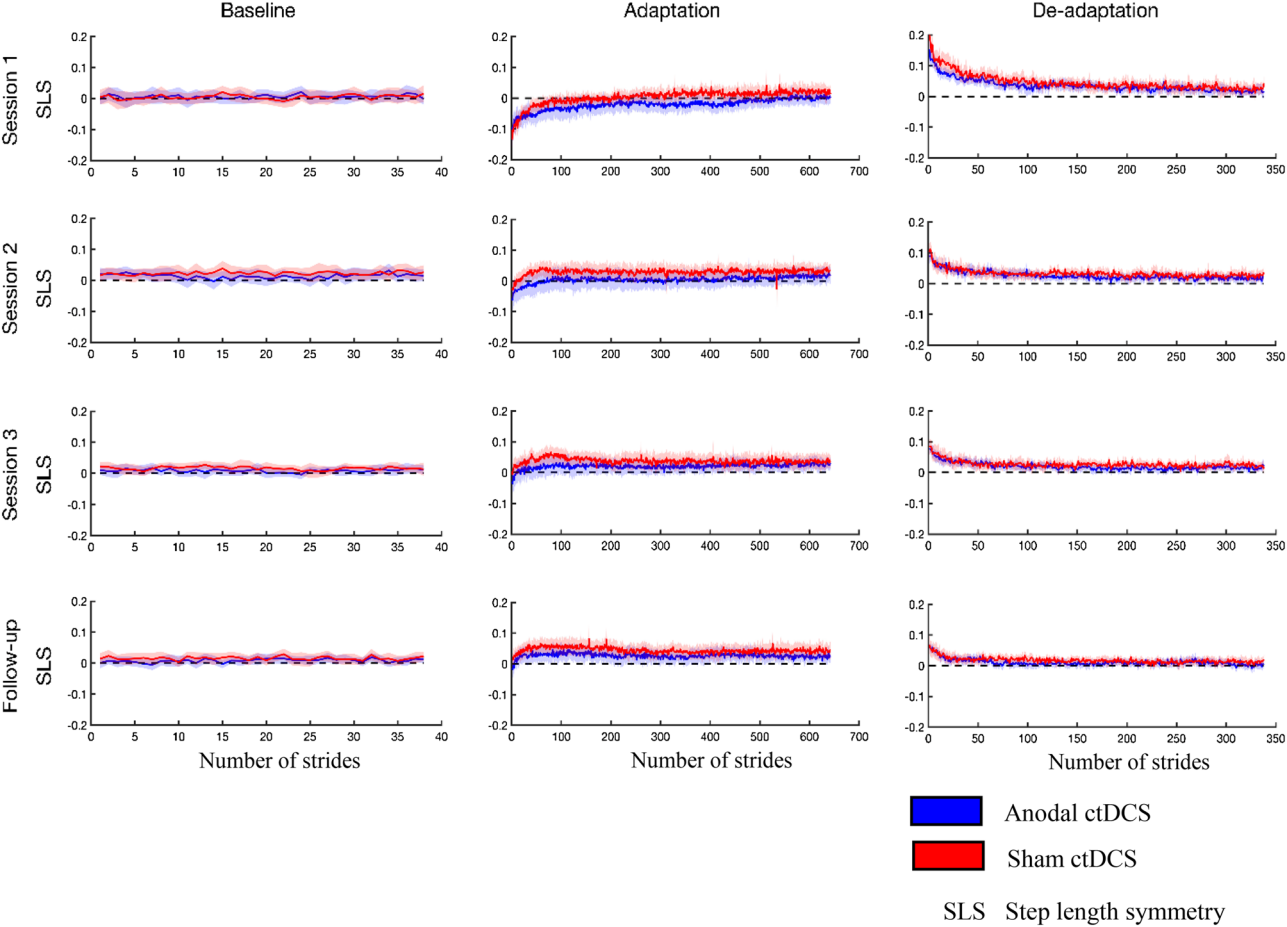
Graphs illustrating the stride-by-stride plots of step length symmetry averaged over all participants in each group across days.

**Figure 6:**
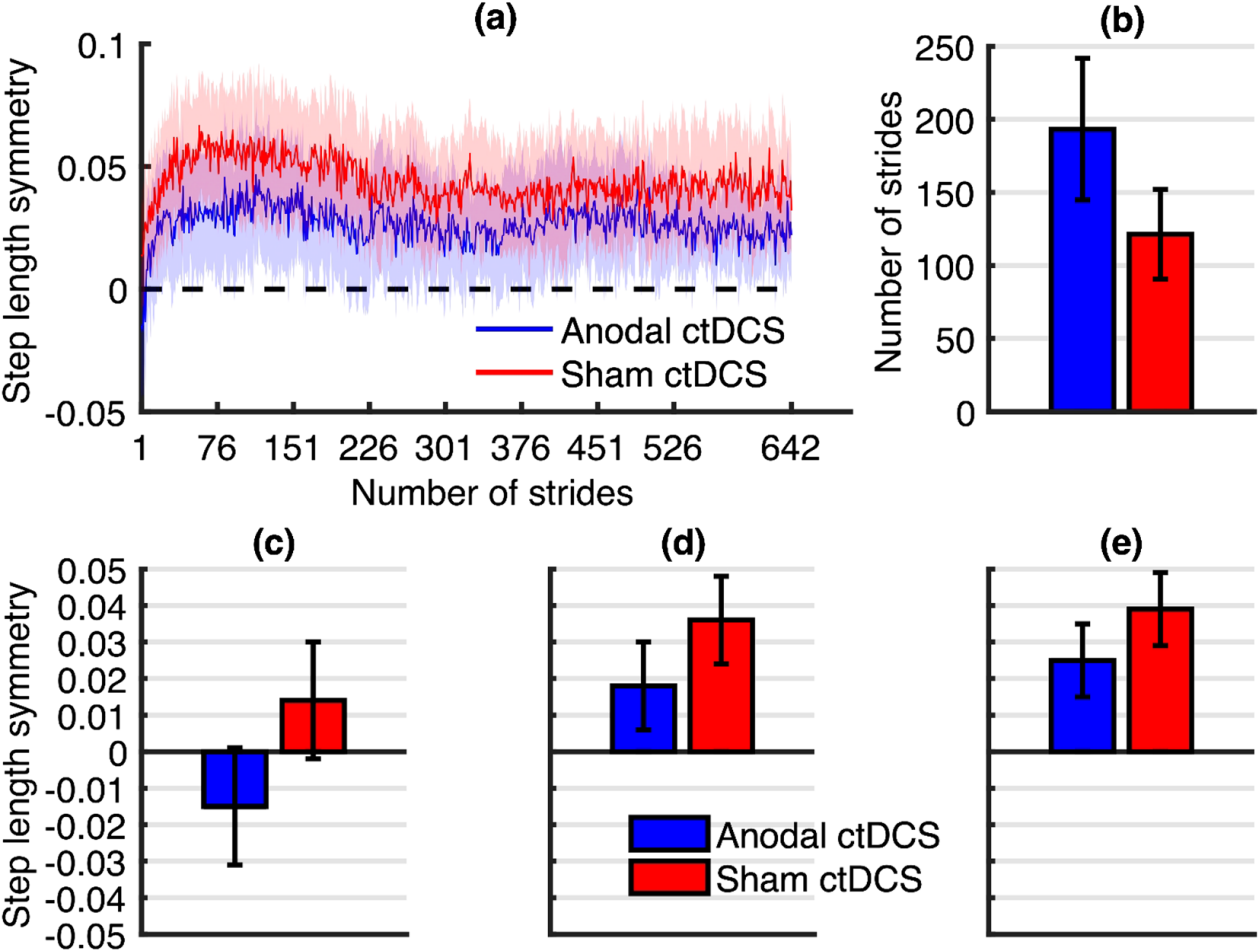
Graphs illustrating the results of the adaptation phase at the follow-up session. (**a**) Stride-by-stride mean step length symmetry plot for the anodal ctDCS group (blue) and sham ctDCS group (red) during the adaptation phase of the follow-up session. Lightly shaded areas indicate a 95% confidence interval. The inset bar graphs indicate mean estimates and standard error from the statistical models for (**b**) the number of strides to steady-state, (**c**) immediate adaptation, (**d**) early adaptation, (**e**) late adaptation.

**Figure 7:**
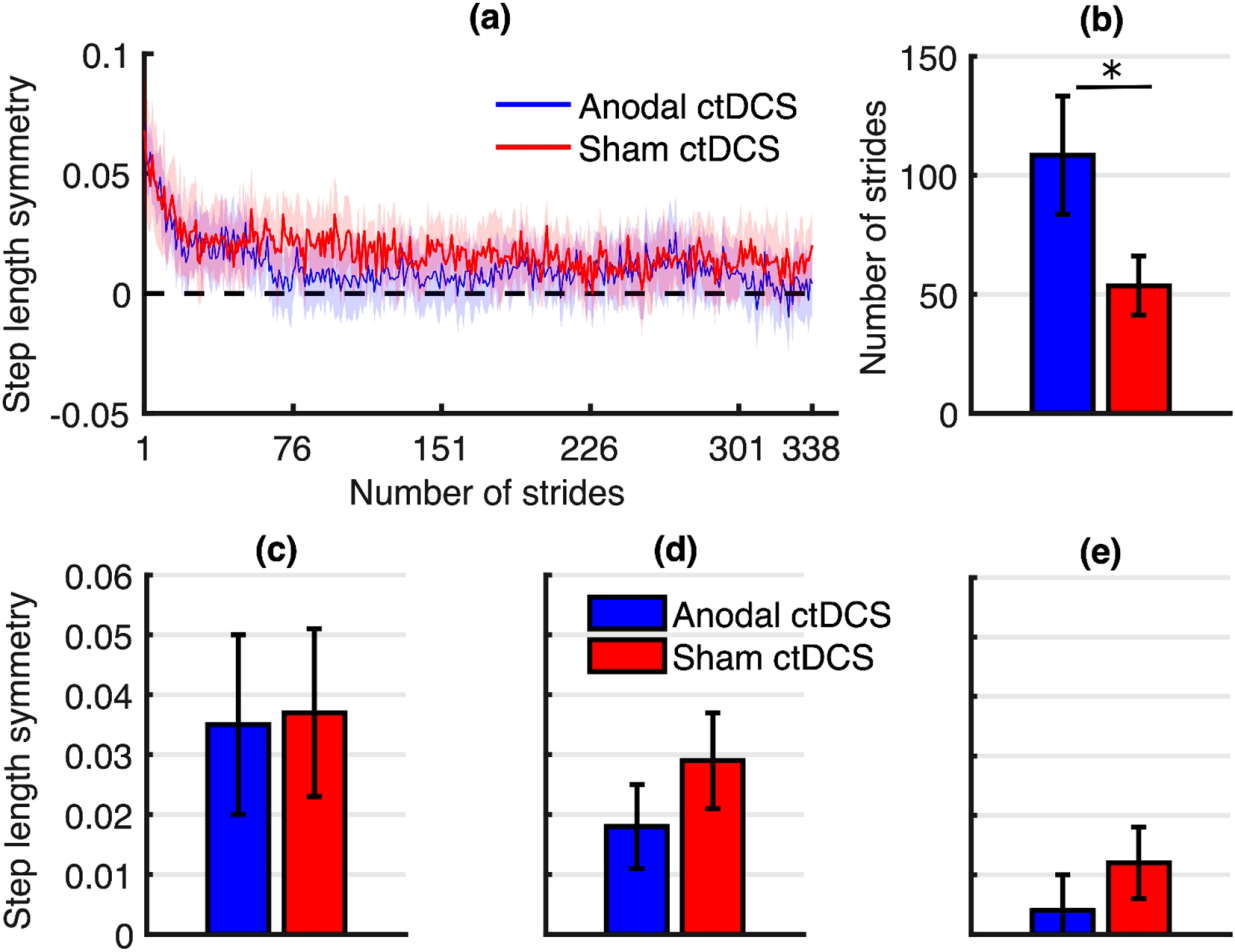
Graphs illustrating the results of the de-adaptation phase at the follow-up session. (**a**) Stride-by-stride mean step length symmetry plot for the anodal ctDCS group (blue) and sham ctDCS group (red) during the de-adaptation phase of the follow-up session. Lightly shaded areas indicate a 95% confidence interval. The inset bar graphs indicate mean estimates and standard error from the statistical models for (**b**) the number of strides to steady-state, (**c**) immediate de-adaptation, (**d**) early de-adaptation, (**e**) late de-adaptation.

**Figure 8:**
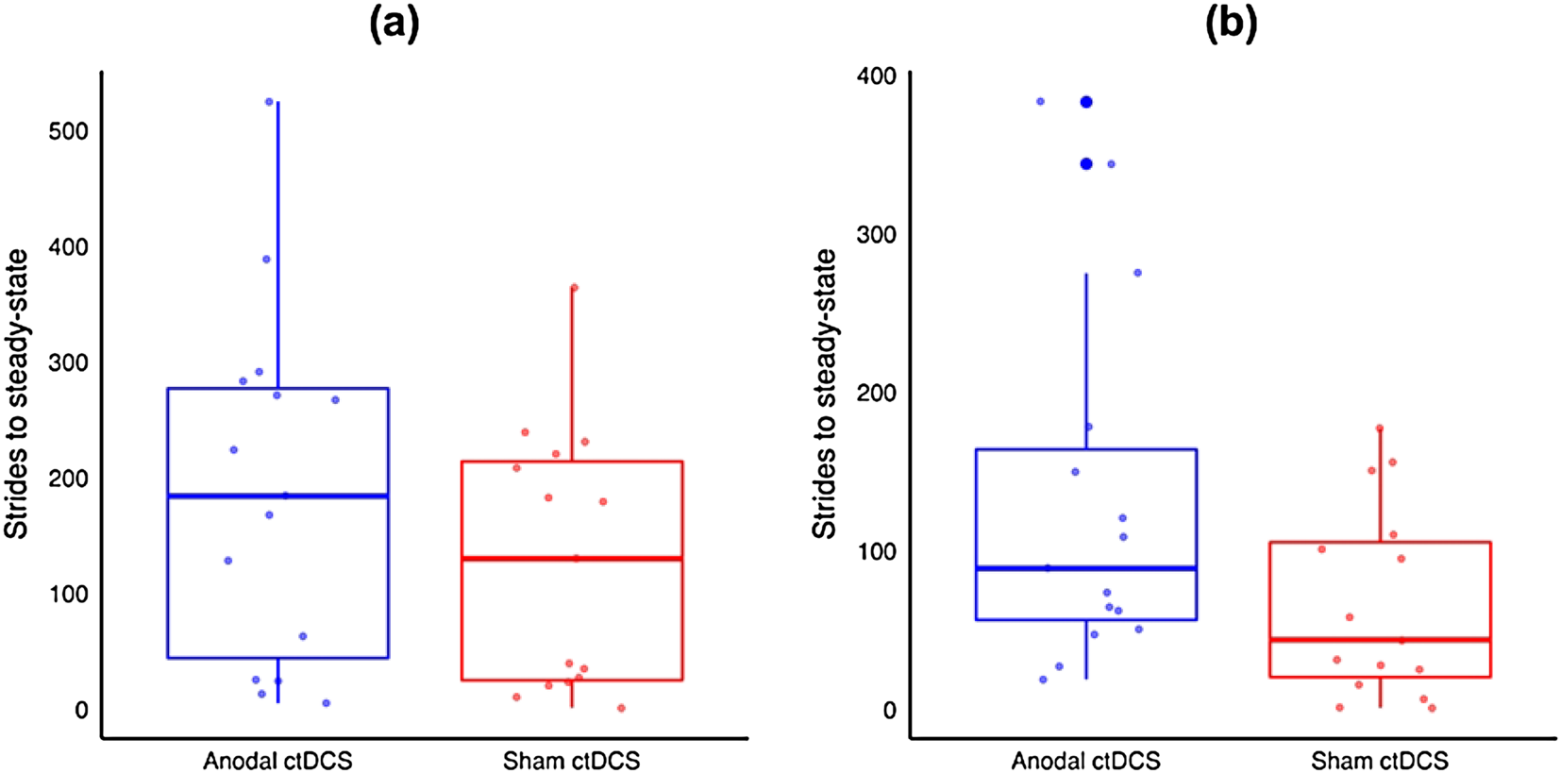
Box and scatter plots for strides to steady-state for (**a**) adaptation and (**b**) de-adaptation phases from the follow-up sessions. The thick horizontal lines represent the median, the hinges of the box plots represent 1st and the 3rd quartile, the whiskers are plotted at a distance of 1.5 times the inter-quartile range, the thick dots represent outliers and the thin dots represent the raw data from individual participants.

The stride-by-stride plots of step length symmetry averaged over all participants in each group across days are represented in Fig. 5. The stride-by-stride plots of step length symmetry averaged over all participants in each group for the follow-up session are represented in Figs. 6 and 7 along with the mean estimates and standard error of strides to steady-state, immediate, early and late adaptation and de-adaptation, respectively. The raw data from individual participants for strides to steady-state is presented in Fig. 8 with box and scatter plots.

The comparison of absolute learning, cumulative effect, consecutive-session effects, and session-specific effects between the anodal ctDCS and sham ctDCS groups during the adaptation and de-adaptation phases is elaborated in Tables 1 and 2. It should be noted that larger estimates in the adaptation phase imply *more* adaptation and *less time taken* to adapt, whereas larger estimates in the de-adaptation phase mean *less* de-adaptation and *more time taken* to de-adapt.

**Table 1:** Contrast estimates based on marginal means for the adaptation phase treatment effects with the standard errors estimated from the statistical models.

Type of effect (session)	Outcome measure	T_X_ ± S.E.	[95% CI]	t[df] or z	*p*
Learning effect (4)	Immediate	− 0.029 ± 0.022	[− 0.073, 0.015]	− 1.353[52.1]	0.182
	Early	− 0.020 ± 0.018	[− 0.056, 0.016]	− 1.050[51.5]	0.299
	Late	− 0.015 ± 0.014	[− 0.043, 0.013]	− 1.081[49.1]	0.285
	STS (ratio)	1.592 ± 0.569	[0.789, 3.210]	1.299	0.194
Cumulative effect (1,3)	Immediate	− 0.039 ± 0.015	[− 0.069, − 0.01]	− 2.586[96.4]	0.011*
	Early	− 0.012 ± 0.013	[− 0.038, 0.014]	− 0.908[91.8]	0.366
	Late	0.015 ± 0.009	[− 0.003, 0.033]	1.729[96.4]	0.087
	STS (ratio)	0.830 ± 0.417	[0.310, 2.220]	− 0.371	0.711
Consecutive-session effect (1,2)	Immediate	− 0.039 ± 0.015	[− 0.069, − 0.01]	− 2.604[96.4]	0.011*
	Early	− 0.012 ± 0.013	[− 0.038, 0.014]	− 0.887[91.8]	0.377
	Late	0.010 ± 0.009	[− 0.008, 0.028]	1.126[96.4]	0.263
	STS (ratio)	0.854 ± 0.428	[0.320, 2.280]	− 0.316	0.752
Consecutive-session effect (2,3)	Immediate	0.0002 ± 0.015	[− 0.030, 0.030]	0.017[96.4]	0.986
	Early	− 0.0003 ± 0.0135	[− 0.027, 0.027]	− 0.019[91.7]	0.985
	Late	0.005 ± 0.009	[− 0.013, 0.023]	0.603[96.4]	0.548
	STS (ratio)	0.972 ± 0.489	[0.363, 2.610]	− 0.056	0.956
Session-specific effect (1)	Immediate	0.013 ± 0.022	[− 0.031, 0.057]	0.581[52.1]	0.564
	Early	− 0.015 ± 0.018	[− 0.051, 0.021]	− 0.821[50.3]	0.415
	Late	− 0.023 ± 0.014	[− 0.051, 0.005]	− 1.694[49.1]	0.097
	STS (ratio)	1.204 ± 0.428	[0.600, 2.420]	0.522	0.602
Session-specific effect (2)	Immediate	− 0.027 ± 0.022	[− 0.071, 0.017]	− 1.231[52.1]	0.224
	Early	− 0.027 ± 0.018	[− 0.063, 0.009]	− 1.457[52.7]	0.151
	Late	− 0.013 ± 0.014	[− 0.041, 0.015]	− 0.968[49.1]	0.338
	STS (ratio)	1.028 ± 0.366	[0.511, 2.070]	0.076	0.939
Session-specific effect (3)	Immediate	− 0.026 ± 0.022	[− 0.070, 0.018]	− 1.219[52.1]	0.228
	Early	− 0.027 ± 0.018	[− 0.063, 0.009]	− 1.472[52.5]	0.147
	Late	− 0.008 ± 0.014	[− 0.036, 0.020]	− 0.579[49.1]	0.565
	STS (ratio)	0.999 ± 0.354	[0.499, 2.000]	− 0.003	0.998

*STS* strides to steady-state, *TX* treatment effect (in actual units i.e. step length symmetry for immediate, early, and late outcome measures; in ratio for strides to steady-state performance; for example, a ratio effect size of 1.592 means that the experimental group has a 59 percent higher mean value compared to the control group for strides to state-state performance).

*S.E.* standard error, *CI* confidence interval, *t* t-statistics, *df* degrees of freedom, *z* z-statistics, *p p* value, * *p < 0.05*

**Table 2:** Contrast estimates based on marginal means for the de-adaptation phase treatment effects with the standard errors estimated from the statistical models.

Type of effect (session)	Outcome measure	T_X_ ± S.E	[95% CI]	t[df] or z	*p*
Learning effect (4)	Immediate	− 0.003 ± 0.014	[− 0.031, 0.025]	− 0.177[84]	0.860
	Early	− 0.012 ± 0.008	[− 0.028, 0.004]	− 1.455[92.4]	0.149
	Late	− 0.008 ± 0.006	[− 0.020, 0.004]	− 1.365[86.4]	0.176
	STS (ratio)	2.024 ± 0.659	[1.069, 3.830]	2.166	0.030*
Cumulative effect (1,3)	Immediate	0.035 ± 0.014	[0.007, 0.063]	2.427[96.4]	0.017*
	Early	0.006 ± 0.009	[− 0.012, 0.024]	0.645[92.7]	0.521
	Late	− 0.002 ± 0.006	[− 0.014, 0.010]	− 0.331[96.4]	0.742
	STS (ratio)	1.287 ± 0.461	[0.638, 2.600]	0.705	0.481
Consecutive-session effect (1,2)	Immediate	0.027 ± 0.014	[− 0.0008, 0.055]	1.864[96.4]	0.065
	Early	0.006 ± 0.009	[− 0.012, 0.024]	0.671[92.8]	0.504
	Late	− 0.002 ± 0.006	[− 0.014, 0.010]	− 0.277[96.4]	0.782
	STS (ratio)	1.220 ± 0.423	[0.617, 2.410]	0.571	0.568
Consecutive-session effect (2,3)	Immediate	0.008 ± 0.014	[− 0.020, 0.036]	0.563[96.4]	0.575
	Early	0.0002 ± 0.009	[− 0.018, 0.018]	− 0.026[93.1]	0.980
	Late	− 0.0003 ± 0.006	[− 0.012, 0.012]	− 0.054[96.4]	0.957
	STS (ratio)	1.056 ± 0.377	[0.525, 2.120]	0.152	0.879
Session-specific effect (1)	Immediate	− 0.044 ± 0.014	[− 0.072, − 0.016]	− 3.015[84]	0.003*
	Early	− 0.009 ± 0.008	[− 0.025, 0.007]	− 1.112[87.6]	0.269
	Late	− 0.006 ± 0.006	[− 0.018, 0.006]	− 1.079[86.4]	0.284
	STS (ratio)	0.756 ± 0.243	[0.403, 1.420]	− 0.872	0.383
Session-specific effect (2)	Immediate	− 0.017 ± 0.014	[− 0.045, 0.011]	− 1.179[84]	0.242
	Early	− 0.003 ± 0.008	[− 0.019, 0.013]	− 0.371[89.8]	0.711
	Late	− 0.008 ± 0.006	[− 0.02, 0.004]	− 1.374[86.4]	0.173
	STS (ratio)	0.922 ± 0.297	[0.490, 1.730]	− 0.253	0.800
Session-specific effect (3)	Immediate	− 0.009 ± 0.014	[− 0.037, 0.019]	− 0.625[84]	0.534
	Early	− 0.003 ± 0.008	[− 0.019, 0.013]	− 0.399[90]	0.691
	Late	− 0.009 ± 0.006	[− 0.021, 0.003]	− 1.431[86.4]	0.156
	STS (ratio)	0.973 ± 0.321	[0.509, 1.860]	− 0.083	0.934

*STS* strides to steady-state, *TX* treatment effect (in actual units i.e. step length symmetry for immediate, early, and late outcome measures; in ratio for strides to steady-state performance; for example, a ratio effect size of 2.02 means that the experimental group has a 102 percent higher mean value compared to the control group for strides to state-state performance).

*S.E.* standard error, *CI* confidence interval, *t* t-statistics, *df* degrees of freedom, *z* z-statistics, *p p* value, * *p < 0.05*

### Adaptation

#### Absolute learning

The linear mixed model analysis revealed that compared to sham ctDCS, anodal ctDCS had no statistically significant effect on the absolute learning of a split-belt treadmill walking task during the adaptation phase, as illustrated by findings for immediate (*p* = 0.18), early (*p* = 0.30) and late adaptation (*p* = 0.29), and strides to steady-state (*p* = 0.19). Absolute learning results are presented in Fig. 6b–e.

#### Cumulative effect

**Figure 9:**
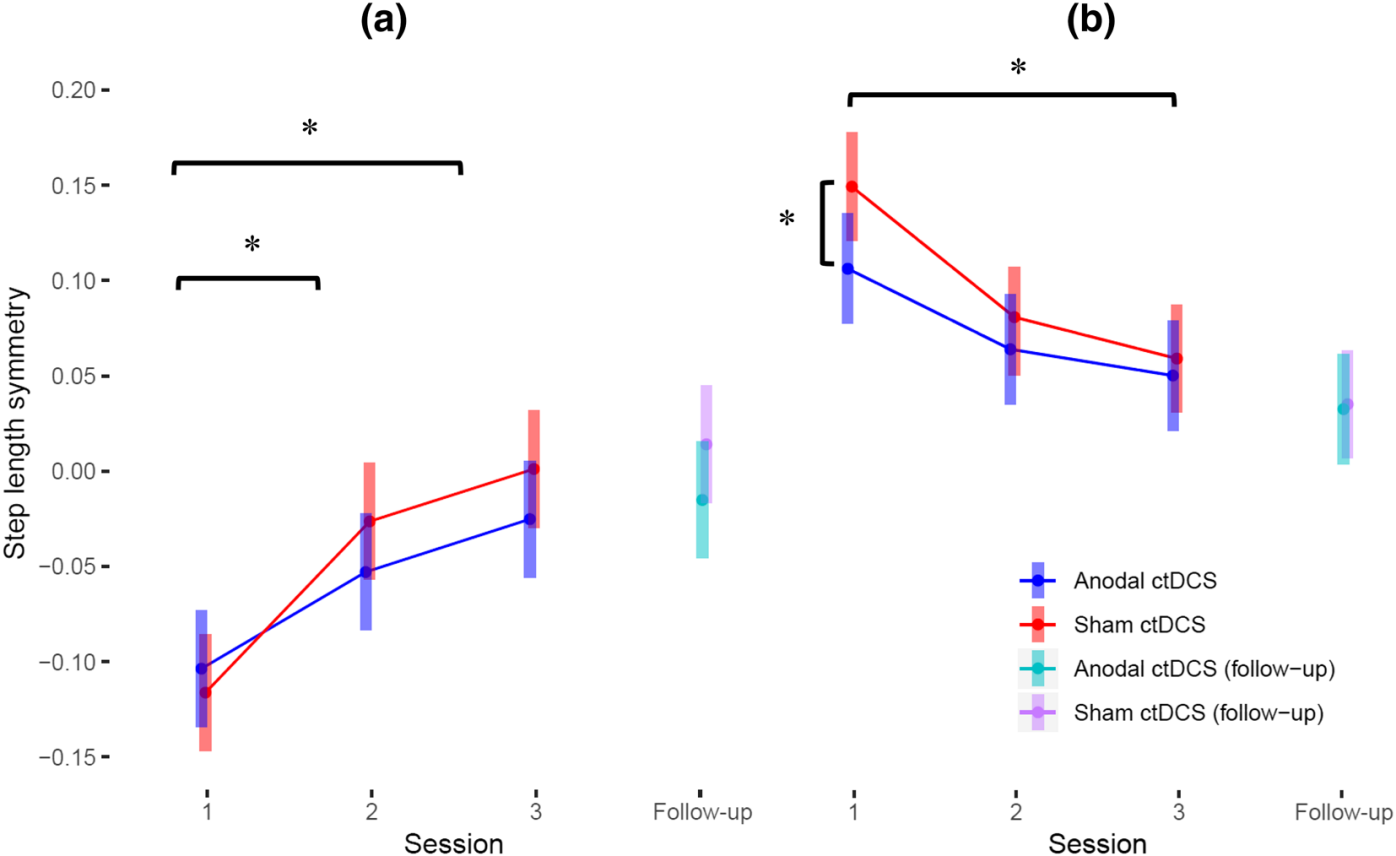
Comparison of mean estimates and 95% confidence intervals between groups over the four sessions for (**a**) immediate adaptation and  (**b**) immediate de-adaptation.

There was a statistically significant cumulative effect on immediate adaptation such that anodal ctDCS caused a smaller change in immediate adaptation across the three intervention sessions (*p* = 0.01, − 0.039, S.E. = 0.015) (see Fig. 9a for immediate adaptation results). However, anodal ctDCS had no statistically significant cumulative effect on early adaptation (*p* = 0.37), late adaptation (*p* = 0.09), or strides to steady-state (*p* = 0.71).

#### Consecutive-session effects

There was a statistically significant consecutive-session effect on immediate adaptation such that anodal ctDCS caused smaller change in immediate adaptation between sessions 1 and session 2 (*p* = 0.011, − 0.039, S.E. = 0.015) but had no effect between sessions 2 and 3 (*p* = 0.99) (see Fig. 9a for immediate adaptation results). However, anodal ctDCS had no statistically significant consecutive-session effect, between sessions 1 and 2 or sessions 2 and 3, on early adaptation (*p* = 0.38, *p* = 0.99), late adaptation (*p* = 0.26, *p* = 0.55), or strides to steady-state (*p* = 0.75, *p* = 0.96).

#### Session-specific effects

As compared to sham ctDCS, anodal ctDCS had no statistically significant session-specific effect on immediate, early, or late adaptation or the strides to steady-state in any of the three intervention sessions (see Table 1 and supplementary file for contrast estimates for treatment effects).

### De-adaptation

#### Absolute learning

As compared to sham ctDCS, anodal ctDCS had no statistically significant effect on the immediate (*p* = 0.86), early (*p* = 0.15), or late de-adaptation (*p* = 0.18). However, there was a statistically significant effect on absolute learning during de-adaptation phase for strides to steady-state such that anodal ctDCS slowed de-adaptation at the follow-up session (*p* = 0.03, 2.024, S.E. = 0.659). Absolute learning results are presented in Fig. 7b–e.

#### Cumulative effect

There was a statistically significant cumulative effect on the immediate de-adaptation (*p* = 0.02, 0.035, S.E. = 0.014) such that anodal ctDCS caused a larger change in immediate de-adaptation across the three intervention sessions (see Fig. 9b for immediate de-adaptation results). However, anodal ctDCS had no statistically significant cumulative effect on early de-adaptation (*p* = 0.52), late de-adaptation (*p* = 0.74), or strides to steady-state (*p* = 0.48).

#### Consecutive-session effects

As compared to sham ctDCS, anodal ctDCS had no statistically significant consecutive-session effect, either between sessions 1 and 2 or sessions 2 and 3, on the immediate de-adaptation (*p* = 0.07, *p* = 0.58), early de-adaptation (*p* = 0.50, *p* = 0.98), late de-adaptation (*p* = 0.78, *p* = 0.96), or strides to steady-state (*p* = 0.57, *p* = 0.88).

#### Session-specific effects

There was a statistically significant session-specific effect on immediate de-adaptation in sessions such that anodal ctDCS caused a greater session-specific immediate de-adaptation in session 1 (*p* = 0.003, − 0.044, S.E. = 0.014) but had no effect on subsequent sessions (see Fig. 9b for immediate de-adaptation results). However, anodal ctDCS had no session-specific effect on the early or late de-adaptation or the strides to steady-state in any of the three intervention sessions (see Table 2 and supplementary file for contrast estimates for treatment effects).

## Discussion

This study investigated the effects of multiple days of anodal ctDCS on learning a split-belt treadmill walking task. Successful rehabilitation outcomes depend on improvements in motor performance which persist beyond the intervention period^44^. Elucidating the effects of anodal ctDCS on motor learning is critical if it is to be used as a rehabilitation tool. It was found that three sessions of anodal ctDCS did not influence motor learning measured during the adaptation phase of a split-belt treadmill task. In contrast to the hypotheses, anodal ctDCS reduced the cumulative and consecutive-session effects on immediate adaptation across three sessions and between the first two sessions of the intervention, respectively. This suggests that anodal ctDCS impairs immediate changes in motor performance during the intervention but does not influence motor learning measured after a delay of one week. Interestingly, during the de-adaptation phase, anodal ctDCS significantly prolonged the training effect without impacting immediate, early or late de-adaptation. This indicates that anodal ctDCS affects the length of time that healthy individuals maintain an adapted walking pattern during the de-adaptation phase. Furthermore, anodal ctDCS induced cumulative immediate de-adaptation across the three sessions of intervention and greater immediate de-adaptation in session 1. These findings suggest that ctDCS may extend the benefits of motor training by enhancing the maintenance of learnt patterns.

To our knowledge, this is the first study to demonstrate that three sessions of anodal ctDCS does not affect the adaptation phase motor learning after a delay of one week as compared to sham ctDCS. One study has investigated the effects of three sessions of anodal ctDCS using a motor skill learning paradigm. In contrast to the findings of our study, the authors reported improved speed-accuracy trade-off in an upper limb skill task after a delay of one week^23^. Our contrasting findings may be explained by the differences in task characteristics. A motor skill task may take weeks and months to master, whereas optimal performance may be achieved within a single day’s practice of a motor adaptation task^45^. This may render motor adaptation tasks subject to ceiling effects in healthy individuals. Differential anodal ctDCS effects with respect to task characteristics have also been noted following a single session of anodal ctDCS where stimulation enhanced gains in motor performance measured up to 48 h after the intervention in motor skill learning^9,10^ but not motor adaptation paradigms^46,47^. The cerebellum’s contribution to motor learning is to a large extent dependent on error-based learning; repeated exposure to the same adaptation task may provide an insufficient stimulus to evoke a cerebellar contribution to motor learning^48^. The importance of the size of the stimulus for error-driven recruitment of the cerebellum is reflected in our results for cumulative and consecutive-session effects of anodal ctDCS which illustrate that anodal ctDCS modulates immediate adaptation but not early or late adaptation. It is likely that there is an insufficient error stimulus in the early or late phases for the effects of ctDCS to be observable^49^. This is also supported by neurophysiological studies which report that activation of the cerebellum depends on the time scale of adaptation^50^ where cerebellar activation decreases over time^5^.

Furthermore, anodal ctDCS modulated immediate adaptation by reducing the cumulative and consecutive-session effect across three sessions and between the first two sessions of the intervention, respectively. The reason for the impaired gains could be related to homeostatic plasticity, where the repetition of tDCS after a break may reverse the expected facilitatory or inhibitory effects resulting in interference with performance^51^. Induction of homeostatic plasticity is dependent on the repetition interval where the second intervention session must be administered during the after-effects of the first session^52^. In a study involving cathodal tDCS over the motor cortex, the authors reported reduced inhibition of cortical excitability when tDCS was delivered 3–24 h after the first intervention session^53^. However, such homeostatic plasticity-induced changes are relatively unexplored in tDCS over the cerebellum. Therefore, investigating cerebellar tDCS-induced cortical excitability and motor performance changes with respect to repetition interval is an important issue for future research.

Anodal ctDCS had no absolute, cumulative, or consecutive-session effect on strides to steady-state performance. This may relate to the role of the cerebellum in the multi-day adaptation process. A recent fMRI study identified neural predictors of adaptability by evaluating the time course of activation over four sessions of a visuomotor adaptation task. Faster adaptation in later sessions was associated with activation of non-cerebellar regions, while slower adaptation was associated with greater activation in the M1-cerebellar motor loop^50^. Therefore, increasing the excitability of the cerebellum with anodal ctDCS may cause slower adaptation as reflected by the cumulative and consecutive-session estimates in this study. Although there was no statistically significant cumulative effect and consecutive-session effect on the strides to steady-state performance, the estimates for anodal ctDCS were larger than sham ctDCS, indicating slower adaptation.

There was no session-specific effect of anodal ctDCS on motor performance or strides to steady-state during the three intervention sessions. In session 1, anodal ctDCS had no effect on immediate adaptation or late adaptation, which is consistent with previous observations^20,46^. However, the results for early adaptation and strides to steady-state are in contrast to those of Jayaram and colleagues^20^ who found an enhanced early adaptation and adaptation rate with a single session of anodal ctDCS. Possible reasons for the inconsistent result could be due to differences in split-belt treadmill protocols and the method of calculating outcomes between the two studies. In our split-belt treadmill protocol, the slow and fast belt speed was set to the individual’s fastest comfortable treadmill walking speed at a ratio of 2:1. In contrast, Jayaram et al. used a fixed speed at the ratio of 3:1 for all participants^20^. We individualised the calculation of early adaptation and rate of adaptation whereas Jayaram et al. used a fixed number of strides to estimate early adaptation (150 strides) and calculated rate by fitting an exponential function to the group data rather than analysing individual data^20^. Inconsistent findings have been reported in previous studies of ctDCS, particularly when a single session of ctDCS is applied during the adaptation tasks^54–56^. The merits of this findings need to be considered in the context of the methodological rigour of the research^18^. However, these findings may highlight the need to understand how the cerebellar structure, within- and between-individual variability, task characteristics or parameters^18,56–58^ may influence the ctDCS effects.

An important finding was that anodal ctDCS slowed the strides to steady-state during the de-adaptation phase after a delay of one week. Enhancing the maintenance of the adapted walking patterns after training is important in rehabilitation^44^. The mechanism of locomotor learning may explain this finding. In a single session of a motor adaptation task, walking pattern adapts to the induced perturbation which when removed results in persistence of adapted walking patterns for several strides as an after-effect. This is represented by immediate de-adaptation in our study. However, with repeated adaptation and de-adaptation, the persistence of the adapted walking pattern decreases and ultimately disappears when newly learned locomotor programs become stored separately from the baseline program, and one can automatically switch between two motor patterns without relying on the trial and error-based learning^14,15^. This was reflected in our study results where we found that anodal ctDCS had a cumulative effect between session 1 and session 3 and enhanced the immediate de-adaptation in session 1. Cumulative effects across three sessions and greater immediate de-adaptation in session 1 reflect decreased reliance on central command calibrations suggesting improvement in the ability of the CNS to predict the optimal locomotor pattern^45^. These results highlight the strength of the study design, which enabled the illustration of the effect of the multiple day intervention protocol on both adaptation and de-adaptation.

## Strengths, limitations, and future directions

This study had a strong research design, consisting of a multi-session, randomised, double-blinded sham-controlled design evaluating a range of outcome measures using robust methods to elucidate the effect of an intervention program on long-term learning. However, some limitations should be considered. We set the slow and fast belt speed based on an individual’s fastest comfortable walking speed on the treadmill^29^. This may not have provided enough challenge to healthy individuals. The two belts moved at a speed ratio of 2:1 which may have caused them to reach their asymptote level faster due to the fact that a smaller speed ratio induces a smaller initial error^59^. Considering that our participants were healthy individuals, both of these factors may have caused a ceiling effect. Future studies may wish to examine how anodal ctDCS effects vary with task difficulty in healthy individuals. Another limitation of this research was that the sample size was estimated based on a previous study that utilised a motor skill task^23^. Therefore, this sample size may not be large enough to see its cumulative effects over multiple sessions in motor adaptation tasks. In the future, the results of this research could be used to help estimate the sample sizes for cumulative effect. Due to the small sample sizes, we must be cautious about the use of linear mixed models, due to the issue of anticonservative inference^60^. This means that results that are of only marginal significance, in particular, need to be interpreted cautiously. Lastly, to quantify motor performance we utilised mean step length symmetry which excludes within participant step length symmetry variance. However, this is line with earlier studies involving split-belt training^15,20,49^. Future studies may consider reporting step length variance as an additional outcome measure.

## Conclusions

Three sessions of anodal ctDCS had no effect on motor learning measured during locomotor adaptation in healthy individuals; in fact, it reduced the cumulative and consecutive-session effect on immediate adaptation. Importantly, three sessions of anodal ctDCS enhanced the maintenance of adapted walking patterns during the de-adaptation phase along with having an immediate and cumulative effect on immediate de-adaptation. Extending the time taken to de-adapt following motor training with anodal ctDCS may have potential therapeutic benefits which warrant further investigation.

## Supplementary information

Supplementary file1
