# Supplementary material for: Cerebellar transcranial direct current stimulation for learning a novel split-belt treadmill task: a randomised controlled trial
Source: Sci Rep. 2020 Jul 16;10:11853. doi: 10.1038/s41598-020-68825-2 (PMC7366632; doi:10.1038/s41598-020-68825-2)

Manuscript Title: Cerebellar transcranial direct current stimulation for learning a novel split-belt treadmill task: A randomised controlled trial.

Nitika Kumari, Denise Taylor, Usman Rashid, Alain C. Vandal, Paul F. Smith, and Nada Signal

Supplementary Figure S1: CONSORT study flow diagram.

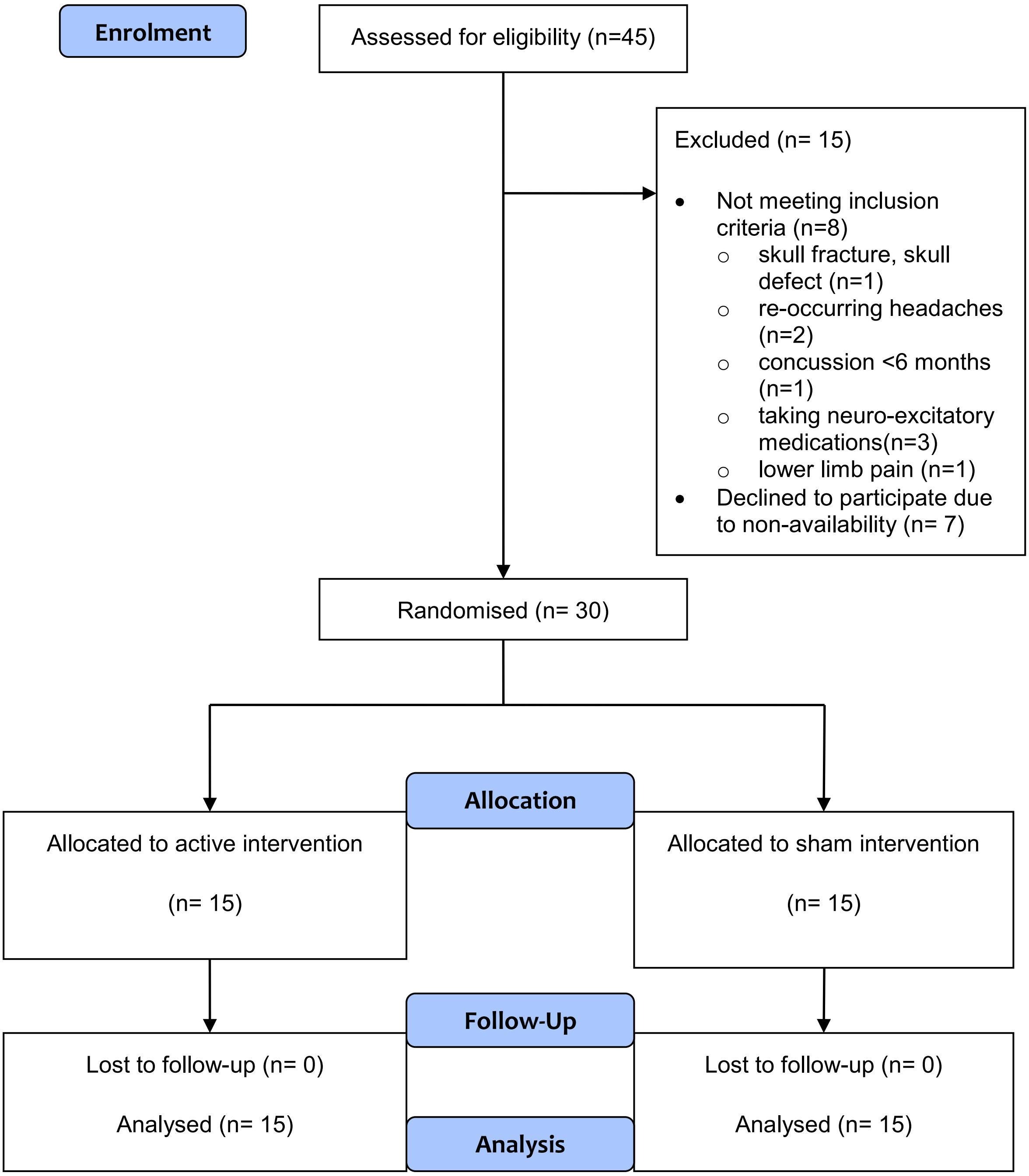

Manuscript Title: Cerebellar transcranial direct current stimulation for learning a novel split-belt treadmill task: A randomised controlled trial.

Nitika Kumari, Denise Taylor, Usman Rashid, Alain C. Vandal, Paul F. Smith, and Nada Signal

Supplementary Table S2: Baseline demographic characteristics.

| | | Active ctDCS | Sham ctDCS |
|---|---|---|---|
| N | | 15 | 15 |
| Age (years) | Mean (SD) | 31.4 (4.14) | 29.8 (8.07) |
| | Range | 23-39 | 21-53 |
| Sex | Male | 9 | 9 |
| | Female | 6 | 6 |
| Height (cm) | Mean (SD) | 172.66 (6.71) | 174.65 (8.74) |
| | Range | 158.8-181.7 | 162.6-193.5 |
| Weight (Kg) | Mean (SD) | 73.81 (11.88) | 76.81 (19.21) |
| | Range | 57.3-92.5 | 53.8-124.5 |
| Leg Dominance | Right | 14 | 14 |
| | Left | 1 | 1 |
| Fastest comfortable walking speed/ Fast belt speed (m/s) | Mean (SD) | 1.60 (0.29) | 1.61 (0.26) |
| | Range | 1.10-2.10 | 1.10-2.10 |

Manuscript Title: Cerebellar transcranial direct current stimulation for learning a novel split-belt treadmill task: A randomised controlled trial.

Nitika Kumari, Denise Taylor, Usman Rashid, Alain C. Vandal, Paul F. Smith, and Nada Signal

Supplementary Figure S3: Contrast estimates based on marginal means for the adaptation phase and de-adaptation phase treatment effects with the 95% confidence interval estimated from the statistical models. Units are in ratio for strides to steady-state; for example, a ratio effect size of 1.592 means that the experimental group has a 59 percent higher mean value compared to the control group for strides to state-state performance.

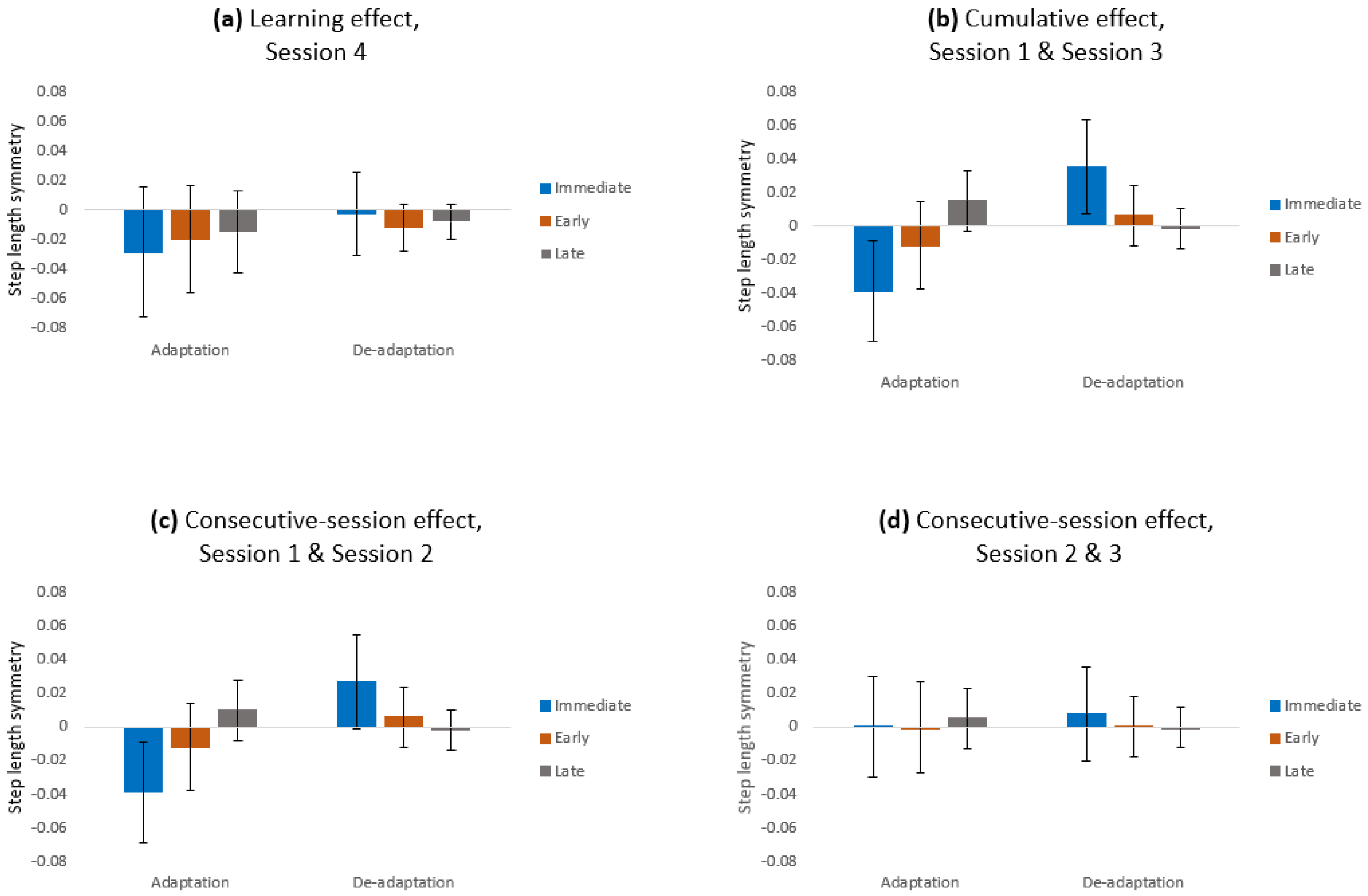

**(e)** Session-specific effect, Session 1

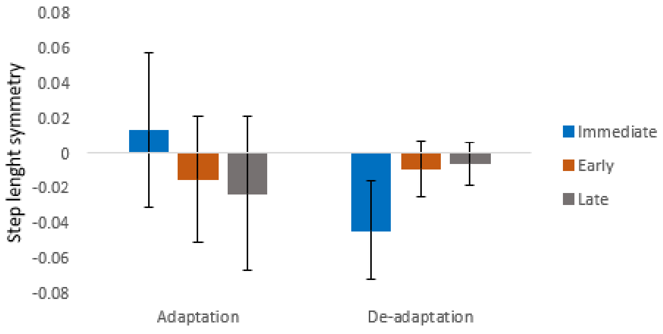


**(f)** Session-specific effect, Session 2

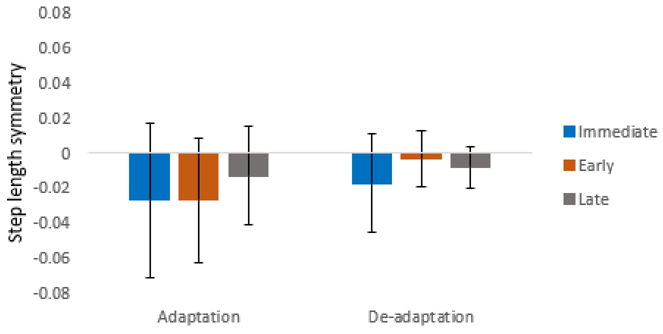


**(g)** Session-specific effect, Session 3

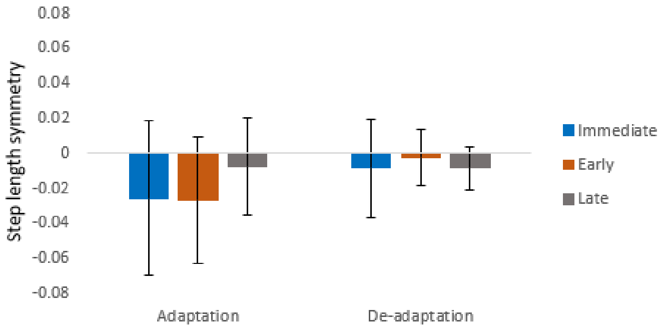


**(h)** Strides to steady-state

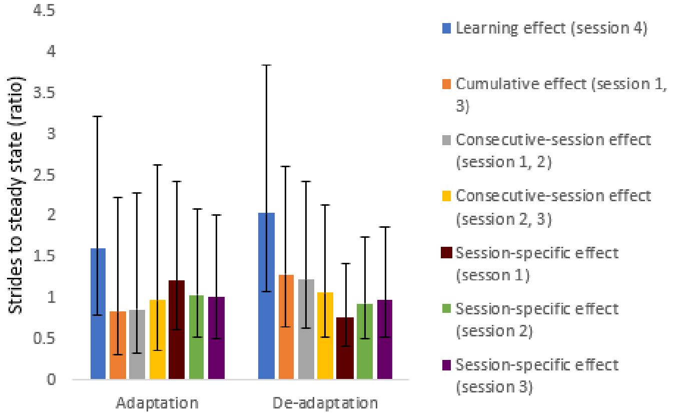

Supplement: Supplementary file 1 — Supplementary file1 [file 41598_2020_68825_MOESM1_ESM.pdf]
